# Identification of telomere-related gene subtypes and prognostic signatures in osteosarcoma

**DOI:** 10.3389/fphar.2025.1545913

**Published:** 2025-02-25

**Authors:** Zhaoguang Song, Wenyan Yu, Xuqing Yin

**Affiliations:** ^1^ Department of West Hospital Orthopaedic Trauma, Zibo Central Hospital, Zibo, China; ^2^ Department of General Family Medicine, Zibo Central Hospital, Zibo, China; ^3^ Department of East Hospital Orthopaedic Trauma, Zibo Central Hospital, Zibo, China

**Keywords:** osteosarcoma, telomeres-related genes, molecular subtypes, prognostic model, tumor microenvironment

## Abstract

**Background:**

Osteosarcoma (OS) is the prevalent primary bone cancer, with a high proclivity for local invasion and metastasis. Previous studies have indicated that telomeres are closely related to prognosis of cancer, but the significance of telomere-related features in OS remains uncertain. Thus, the goal of this work is to identified telomere-related subtypes based on the telomere-related genes (TRGs).

**Methods:**

The data of OS was collected from TARGET and Gene Expression Omnibus databases. Firstly, we identified the subtypes mediated by TRGs in OS. Subsequently, we analyzed the immune characteristics of telomeres-related subtypes in OS. Moreover, we built a telomere-related signature via univariate and LASSO Cox regression analyses, and analyzed the correlation of telomere-related signature with TME. Finally, we analyzed the expression of hub TRGs in OS.

**Results:**

We discovered that TRGs could distinguish OS patients into two telomeres-related subtypes (C1 and C2). The survival rate of OS patients in C2 was inferior to that of patients in C1. The scores of stromal, immune and ESTIMATES were observably increased, and tumor purity was decreased in C1 subtypes compared to C2 subtypes. Differentially expressed genes between C1 and C2 were highly enriched in immune-related pathways. Moreover, C1 and C2 subtypes had different immune characteristic. Furthermore, a telomere prognostic model including six genes (*PDK2*, *PPARG*, *MORC4*, *SP110*, *TERT* and *MAP3K5*) was established to predict the prognosis of OS patients. High-risk group was correlated with inferior prognosis of OS patients, and risk score model was correlated with TME. Finally, we discovered that expression of *PDK2*, *PPARG*, *MORC4*, *SP110*, *TERT* and *MAP3K5* was significantly decreased in OS cells.

**Conclusion:**

In conclusion, our study has uncovered the importance of TRGs in defining distinct subtypes of OS with different survival outcomes and immune contexts. The telomere-related signature we developed may serve as a valuable tool for prognosis prediction and could inform future therapeutic strategies targeting the TME in OS.

## 1 Introduction

Osteosarcoma (OS) is a type of bone cancer that originates from primitive bone-forming cells (Moore and Luu, 2014). It is the most common form of primary bone cancer, primarily affecting teenagers and young adults (Lee et al., 2021). The standard treatment approach for OS typically involves surgical intervention to remove the tumor, followed by chemotherapy aimed at eradicating any residual cancer cells (Gill and Gorlick, 2021). OS often manifests with symptoms such as bone pain, swelling, and restricted movement. The disease exhibits a strong tendency for local invasion and early metastasis to the lung parenchyma, resulting in a poor prognosis and a significant rate of disability (Yang et al., 2020; Sheng et al., 2021; Yui et al., 2022). Lung metastasis impacts nearly half of all OS patients, with 30%–50% succumbing to complications related to pulmonary metastasis (Huang et al., 2009), and approximately 15%–20% of OS patients presenting with lung metastasis at initial diagnosis (Damron et al., 2007; Miller et al., 2013). Furthermore, the complexity and instability of the genome significantly influence the treatment outcomes of OS (Wu and Livingston, 2020). Thus, it is imperative to prioritize the optimization of early diagnosis, treatment, and prognosis of OS from a molecular genetic perspective.

Telomeres, which are nucleoprotein complexes located at the termini of linear chromosomes, play a pivotal role in preserving the structural integrity of these chromosomes (Riethman, 2008). In healthy human cells, telomere length gradually decreases with each cycle of cell division, acting as a crucial barrier against unchecked proliferation and malignant transformation (Yuan et al., 2020). In cancer cells, telomere length is maintained either through the activation of telomerase or alternative lengthening of telomeres (ALT), enabling their ceaseless proliferation; however, their telomeres are typically shorter than those in normal counterparts (Harley, 2008; Shay and Wright, 2019). Telomere shortening may exert two distinct effects on cancer development. Firstly, it may serve a tumor-suppressive role by inhibiting cell growth. Secondly, telomere shortening can lead to significant genome instability, thereby promoting cancer progression (Li et al., 2022). A genetic predisposition toward longer leukocyte telomere length has been identified as a risk factor for acute lymphoblastic leukemia, OS, and neuroblastoma (Walsh et al., 2016). Females with short telomeres may be particularly predisposed to OS (Mirabello et al., 2011). Knockdown of Pin2/TRF1 interacting protein X1 (PinX1) in OS cell lines results in telomere shortening, increased apoptosis, and heightened sensitivity to ionizing radiation (Li et al., 2015). In telomerase-negative OS cells, deletion of the telomere-binding protein tripeptidyl peptidase 1 (TPP1) causes telomere shortening and increases apoptosis and radiation sensitivity (Qiang et al., 2014). Therefore, in OS, telomere shortening plays a significant role in suppressing tumor progression.

Previous studies have primarily concentrated on analyzing telomere length in relation to cancer and its significance for patient prognosis. However, the potential influence of telomere-related genes (TRGs) on the prognosis of OS patients has not been extensively investigated. Therefore, we employed bioinformatics methods to identify two telomere subtypes in OS and assessed the immune cell infiltration and prognostic factors associated with each subtype. Furthermore, we developed a telomere signature based on key TRGs in OS to predict prognosis and explore the potential implications of this telomere signature in the immune landscape of OS.

## 2 Materials and methods

### 2.1 Data acquisition

The mRNA expression profile data of 84 OS patients with complete survival information were extracted from the Therapeutic Applied Research to General Effective Treatments (TARGET) database (https://ocg.cancer.gov/programs/target). The clinicopathological features of these 84 patients are presented in Table 1. Additionally, the GSE16091 and GSE39055 datasets were downloaded from the Gene Expression Omnibus (GEO) database (https://www.ncbi.nlm.nih.gov/geo/). The GSE16091 dataset includes data from 34 OS patients, while the GSE39055 dataset comprises data from 37 OS patients, both with complete survival information. The data for GSE16091 and GSE39055 were obtained using the Affymetrix Human Genome U133A Array and the Illumina HumanHT-12 WG-DASL V4.0 R2 expression beadchip platforms, respectively. Furthermore, the GSE16088 and GSE36001 datasets were also downloaded from the GEO database. The GSE16088 dataset contains chip sequencing data from six normal tissues and 14 OS tissues, whereas the GSE36001 dataset includes data from 4 normal bone tissues, 2 normal bone cells, and 19 OS cells. The data for GSE16088 and GSE36001 were obtained using the Affymetrix Human Genome U133A Array and the Illumina Human-6 v2.0 expression beadchip platforms, respectively.

**TABLE 1:** Clinicopathological characteristics of osteosarcoma patients from TARGET database.

Characteristics		Patients (N = 84)	
		NO.	%
Gender	Female	37	44%
	Male	47	56%
Age	≤14(Median)	44	52%
	>14(Median)	40	48%
Grade	I/II	19	23%
	III/IV	16	19%
Survival Time	Long(>5 years)	28	33%
	Short(<5 years)	57	68%
OS status	Dead	27	32%
	Alive	57	68%

### 2.2 Differential expression and functional enrichment analyses

The differential expression analysis was conducted using the “limma” package in R language (version 4.1.0, as noted below). To identify differentially expressed genes (DEGs) between the two groups, criteria of |Log2FC| > 1 and P. adjust <0.05 were employed for filtering. Subsequently, enrichment analyses for the Kyoto Encyclopedia of Genes and Genomes (KEGG) and Gene Ontology (GO) were carried out on the DEGs, with GO encompassing biological processes, molecular functions, and cellular components. This analysis utilized the “clusterProfiler” utility package in R (Yu et al., 2012). Significantly enriched pathways were determined using a threshold of P < 0.05.

R software was applied to calculate the enrichment score in the GSVA gene set. The pathways and molecular mechanisms were calculated using “c2. cp.kegg.v7.4. symbols.gmt” in MSIGDB.

### 2.3 Immune analysis

Earlier research identified 28 different types of genes associated with immune cells (Lin et al., 2022). By utilizing the expression levels of these genes, the presence of immune cell infiltration in the samples was forecasted through ssGSEA. Consequently, an infiltration profile of immune cells was obtained. The immune score for the samples was computed using the “estimate” function package (https://R-Forge.R-project.org/projects/estimate/).

### 2.4 Least absolute shrinkage selection operator (LASSO) cox regression analysis

Utilizing the DEGs, we performed a univariate Cox regression analysis to identify genes associated with prognosis, applying a significance threshold of P < 0.05. Subsequently, we employed LASSO Cox regression analysis via the “glmnet” package in R to further refine the selection of key prognosis-related genes. Using these pivotal genes, the risk score for each sample was computed according to the following formula:

$$Risk\;score=\sum_{i=1}^{n}\text{Coe}f_{i}\times X_{i}$$

Coefi: the risk coefficient calculated by the lasso Cox model for each factor, Xi: the expression of gene. Next, the value of Risk score was analyzed using R packages survival, survminer and two-sided log rank tests. OS patients were subsequently divided into low- and high-risk groups based on the median of their risk score.

### 2.5 Protein-protein interaction (PPI) network

The STRING (https://string-db.org/) database was employed to create a PPI network. Hub genes were identified within the PPI network utilizing the MCODE plug-in algorithm available in Cytoscape, followed by ClueGO for enrichment analysis and visualization. Interactions among proteins were examined using GeneMANIA (http://www.genemania.org). This advanced tool assists in forming hypotheses regarding gene functions through detailed analyses of gene lists and the effective prioritization of genes for functional testing. Leveraging a broad range of genomic and proteomic datasets, GeneMANIA adeptly identifies genes exhibiting similar functions based on a specified query gene list. Furthermore, GeneMANIA offers essential insights by displaying weights that show the predictive importance of each chosen dataset relative to the query gene.

### 2.6 Survival analysis

The patient’s overall survival was assessed through the use of the “survival” and “survminer” packages in R (https://CRAN.R-project.org/package=survival). Differences in survival rates among various groups were evaluated utilizing the log-rank test.

### 2.7 Cell culture

The osteoblast cell line hFOB1.19 was obtained from the Cell Bank of the Committee on Type Culture Collection, which was part of the Chinese Academy of Sciences located in Shanghai, China. Three OS cell lines, specifically HOS, U2OS, and Saos-2, were sourced from iCell Bioscience Inc., also based in Shanghai, China. hFOB1.19 cells were maintained in a specialized complete culture medium (designated as MD38 from the Type Culture Collection Committee of the Chinese Academy of Sciences) at a temperature of 34°C under a 5% CO_2_ atmosphere. The HOS, U2OS, and Saos-2 cell lines were each cultured in their respective media: MEM for HOS and McCOY’s 5A (for both U2OS and Saos-2), with each medium enriched with 10% FBS and 1% P/S, and incubated at 37°C with 5% CO_2_.

### 2.8 qRT-PCR assay

Total RNA was isolated using TRIzol (DP424, Tiangen Biotech Co., Ltd., Beijing, China). Reverse transcription was carried out using the Evo M-MLV Reverse Transcription Premix Kit (AG11728, Accurate Biology, Changsha, China). Following this, the qRT-PCR assay was performed using the SuperStar Universal SYBR Master Mix (CW3360M, Jiangsu Cowin Biotech Co., Ltd., Jiangsu, China) on a Real-time fluorescence quantitative PCR instrument (SLAN-96S, Shanghai Hongshi Medical Technology Co., Ltd., Shanghai, China). The primer sequences can be found in Table 2. The protocol included a pre-denaturation step at 95°C for 30 s, followed by 40 cycles of 95°C for 10 s and 60°C for 30 s. GADPH served as the internal control. The expression levels of mRNA were calculated using the 2^−ΔΔCT^ method (Jiang et al., 2020; Xue et al., 2024).

**TABLE 2:** Primer sequences for RT-PCR.

Genes	Forward primer (5′-3′)	Reverse primer (5′-3′)	Product length (bp)
PDK2	ATG​AAA​GAG​ATC​AAC​CTG​CTT​CC	GGC​TCT​GGA​CAT​ACC​AGC​TC	82
PPARG	ACC​AAA​GTG​CAA​TCA​AAG​TGG​A	ATG​AGG​GAG​TTG​GAA​GGC​TCT	100
MORC4	CAT​CGC​GGA​GCT​GCT​AGA​TAA	TCC​ATC​ATC​GGT​AAA​GGT​CAA​AC	112
SP110	CCT​ATG​CCA​TAC​ACA​AGC​CAT​T	CCT​CTC​CAG​TTG​GGT​GAG​AAT	152
TERT	CCG​ATT​GTG​AAC​ATG​GAC​TAC​G	CAC​GCT​GAA​CAG​TGC​CTT​C	99
MAP3K5	CTG​CAT​TTT​GGG​AAA​CTC​GAC​T	AAG​GTG​GTA​AAA​CAA​GGA​CGG	120
GAPDH	GAA​GGT​GAA​GGT​CGG​AGT​C	GAA​GAT​GGT​GAT​GGG​ATT​TC	172

### 2.9 Statistical analysis

All statistical analyses were performed using R software (Version 4.1.0). The difference between groups was determined by Wilcoxon rank sum test. Multivariate Cox regression proportional hazards model was used to investigate the impact of risk score and clinicopathological characteristics on overall survival of patients. Statistical significance was set at p < 0.05.

## 3 Results

### 3.1 Identification of telomere subtypes in OS

**FIGURE 1:**
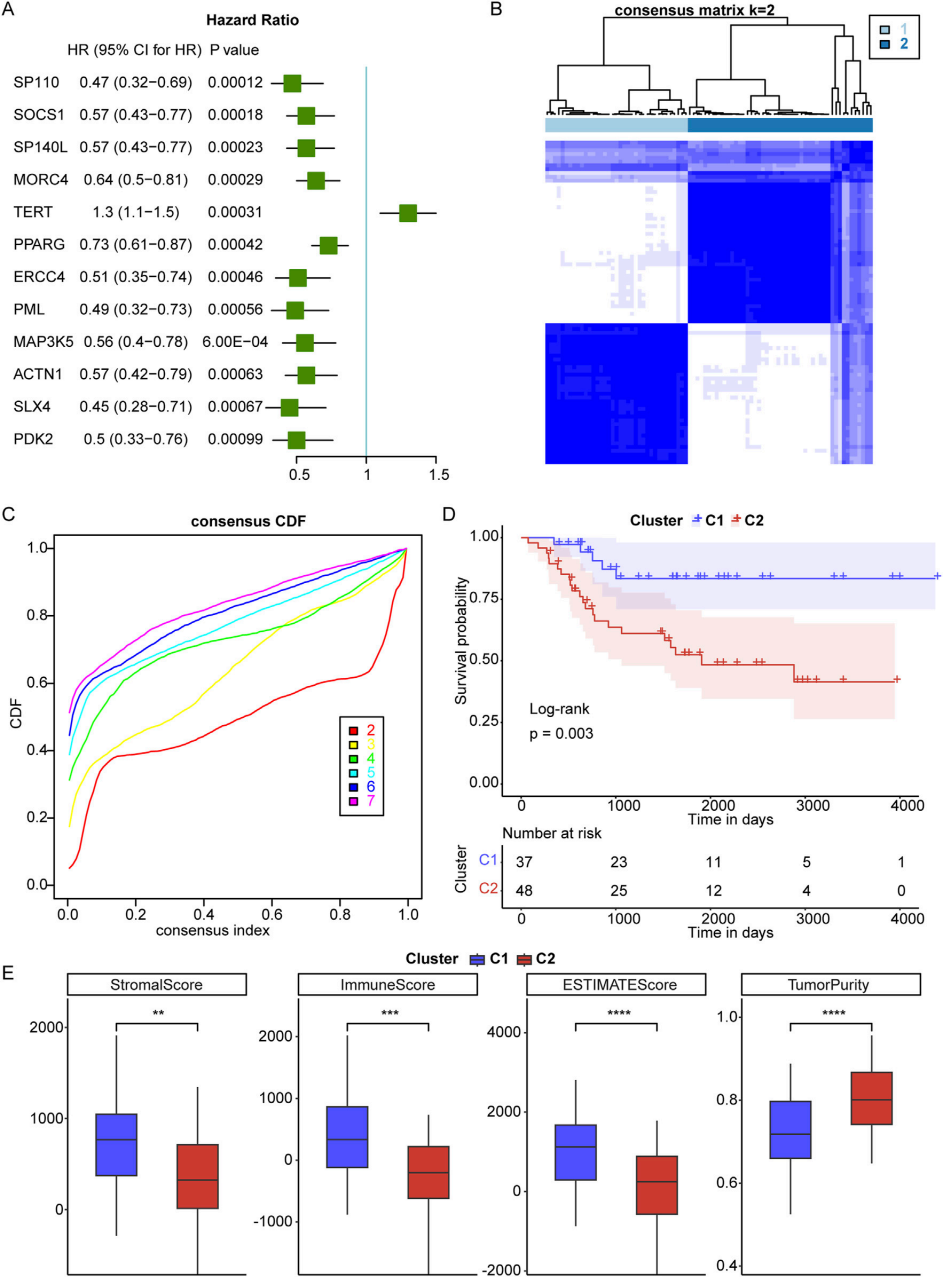
Identification of telomere subtypes in osteosarcoma (OS). **(A)**, Univariate Cox regression analysis was used to screen telomere-related genes that correlated with prognosis of OS. HR means Hazard ratio; 95% CI means 95% confidence interval. **(B)**, Clustering heatmap of prognosis-related telomere genes in OS. **(C)**, Consensus Cumulative Distribution Function (CDF) plot under k = 2–7, where the number of k represents the number of groups after unsupervised clustering. **(D)**, The survival rate of OS patients in C1 and C2 subtypes. **(E)**, The stromal, immune and ESTIMATES scores and tumor purity in C1 and C2 subtypes. **p < 0.01, ***p < 0.001, ****p < 0.0001.

A total of 2086 TRGs were obtained from TelNet (Li et al., 2022). To identify a telomere-relevant signature for OS, these 2086 TRGs were subjected to univariate Cox regression analysis using the TARGET-OS dataset. This analysis revealed 400 TRGs that were significantly associated with the prognosis of OS patients (Figure 1A, p < 0.001). In a cohort of 84 OS samples, we examined the expression of these 400 TRGs through unsupervised clustering, which revealed a significant distinction between two subgroups at k = 2. This suggests that OS patients can be classified into C1 and C2 subtypes (Figures 1B, C). The C1 subtype exhibited a distinct survival advantage, while patients in the C2 subtype demonstrated relatively unfavorable survival outcomes (Figure 1D). Gender and age did not differ significantly between the two clusters, while the pathological stage showed significant differences between the two clusters (Table 3). Furthermore, we analyzed the tumor microenvironment (TME) in the C1 and C2 subtypes using the ESTIMATE algorithm. The stromal, immune, and ESTIMATE scores were significantly increased, and tumor purity was decreased in the C1 subtype compared to the C2 subtype (Figure 1E). These findings indicate that the survival rate of OS patients in the C2 subtype is inferior to that of patients in the C1 subtype.

**TABLE 3:** Clinical characteristics of two clusters.

Characteristic	Number (%)		p-value
	Cluster1 (N = 36)	Cluster2 (N = 48)	
Gender			
Female	18 (21.4%)	19 (22.6%)	0.7835
Male	18 (21.4%)	29 (34.5%)	
Age			
≤14	20 (23.8%)	24 (28.6%)	0.8158
>14	16 (19%)	24 (28.6%)	
Stage			
Stage I-Stage II	5 (6.0%)	14 (16.7%)	0.0441
Stage III-Stage IV	9 (10.7%)	7 (8.3%)	
Unknown	22 (26.2%)	27 (32.1%)	

### 3.2 Immune cell infiltration between telomere subtypes

**FIGURE 2:**
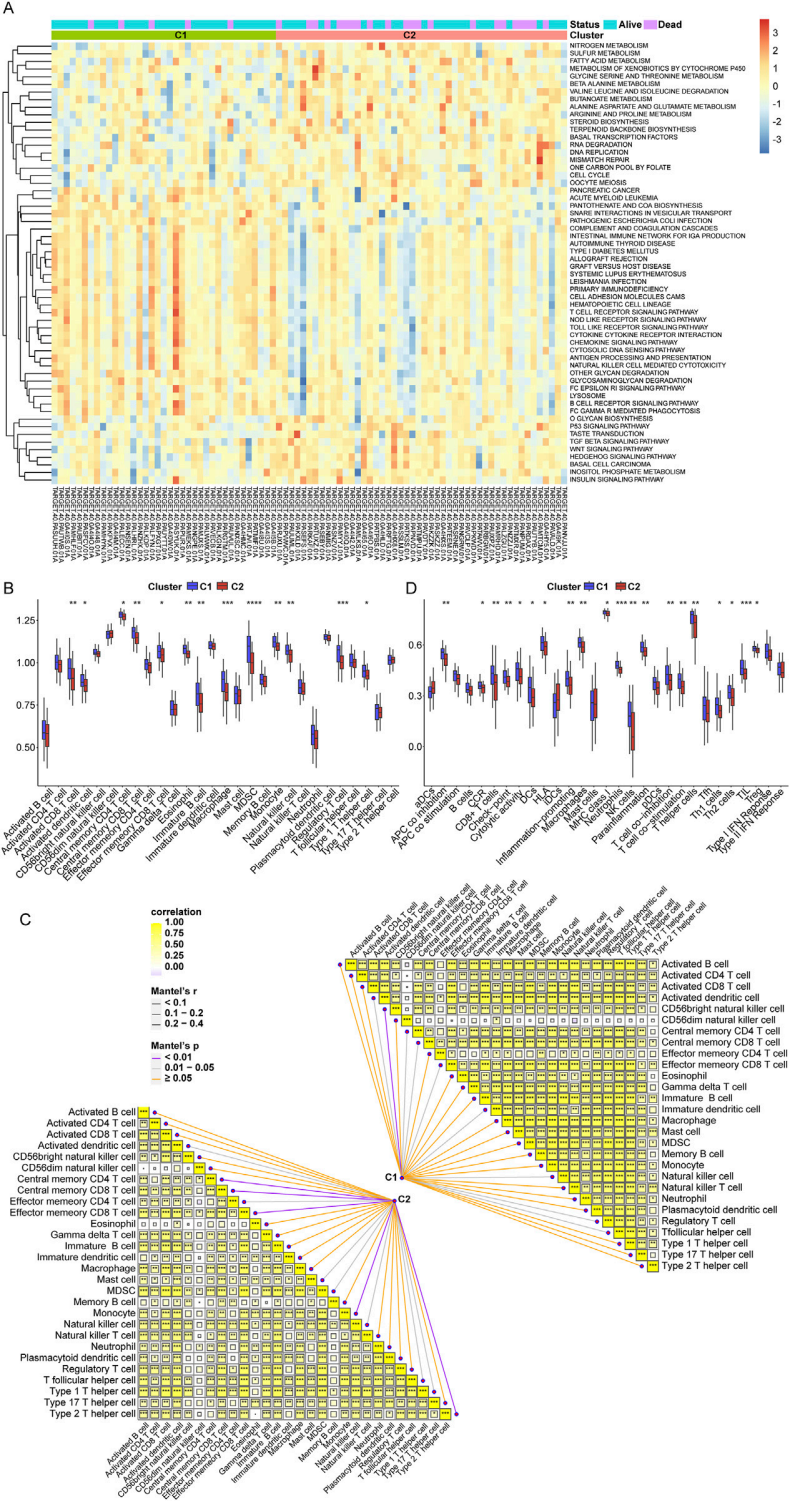
Immune cell infiltration between telomere subtypes. **(A)**, The results of gene set variation analysis (GSVA) analysis. **(B)**, The immune cell infiltration of each sample in C1 and C2 subtypes. **(C)**, The correlation of C1 and C2 subtypes with immune cells. **(D)**, The scores of 16 immune cells and 13 immune related functions in C1 and C2 subtypes. *p < 0.05, **p < 0.01, ***p < 0.001, ****p < 0.0001.

To further investigate the correlation between telomeres and immunity, we conducted GSVA to enrich the different pathways between the C1 and C2 subtypes. In contrast to C1, which demonstrates a distinct prognostic advantage, various immune-related signaling pathways were notably downregulated in C2 (Figure 2A). The infiltration of the TME between the C1 and C2 subtypes was assessed using ssGSEA (Figure 2B). Among 28 immune cells, 13 were significantly enriched in C1, including activated CD8^+^ T cells and activated dendritic cells. Furthermore, we found that the C1 subtype was significantly associated with a greater number of immune cells compared to the C2 subtype (Figure 2C). Additional analyses of the scores for 16 immune cells and 13 immune-related functions in the C1 and C2 subtypes revealed that 10 immune cell scores and 10 immune-related function scores were significantly different between the two subtypes (Figure 2D; Supplementary Table S1). These results suggest that the telomere subtypes of OS exhibit distinct immune characteristics.

### 3.3 Comprehensive analysis of DEGs in telomere subtypes

**FIGURE 3:**
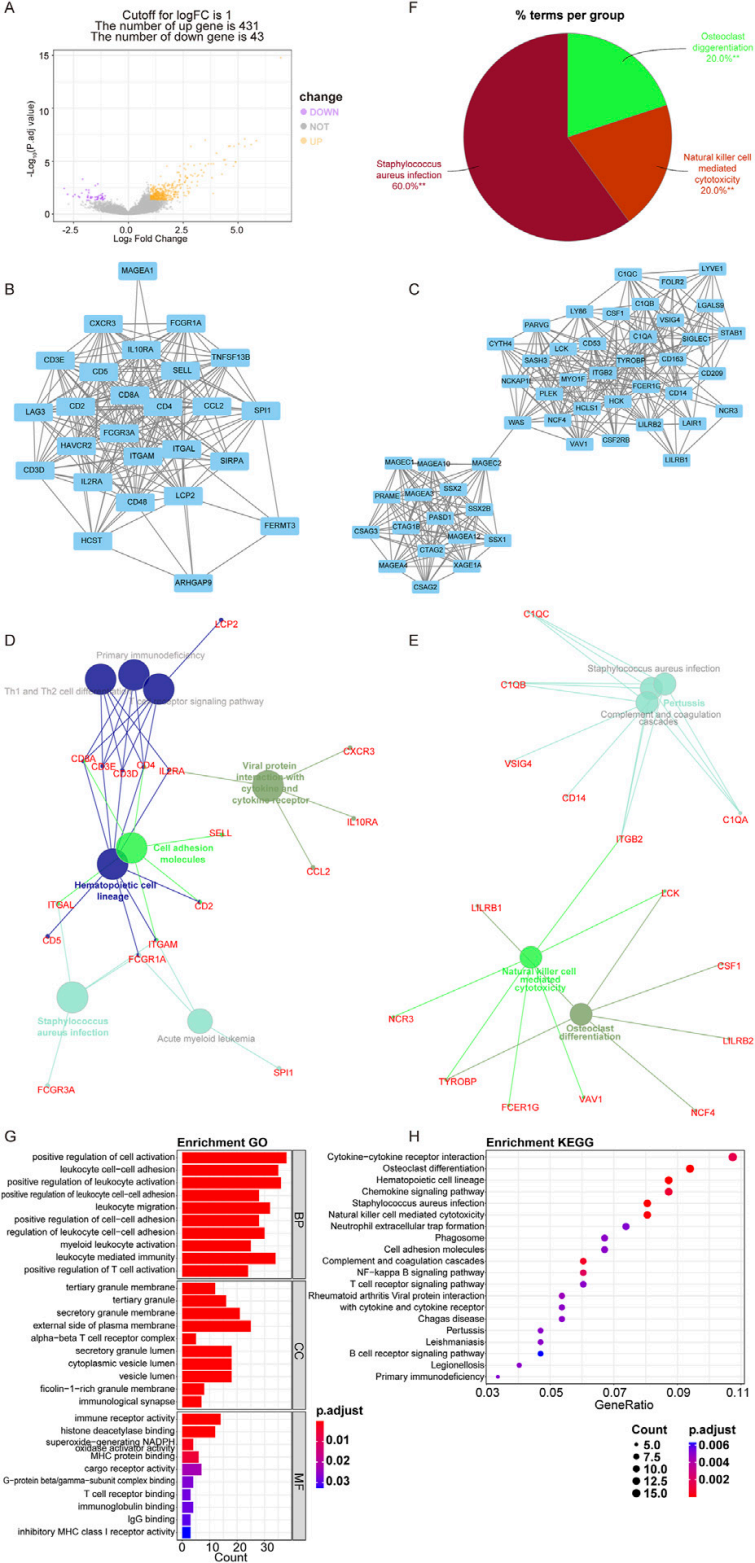
Comprehensive analysis of DEGs between telomere subtypes. **(A)** The differentially expressed genes (DEGs) between C1 and C2 subtypes. The horizontal axis is fold differentially expressed (log2FC). **(B, C)**, Key modules in PPI selected according to molecular complex detection. **(D–F)** ClueGo enrichment results of DEGs between C1 and C2 subtypes (**p < 0.01). **(G)**, Top 10 significantly enriched Gene Ontology terms. BP, biological process; CC, cellular component; MF, molecular function. **(H)**, Top 20 significantly enriched Kyoto Encyclopedia of Genes and Genomes pathways.

Furthermore, we identified DEGs between the C1 and C2 subtypes to further investigate the impact of telomere subtypes on patients with OS. A total of 474 DEGs were identified between the C2 and C1 subtypes, comprising 431 upregulated genes and 43 downregulated genes (C1 vs C2) (Figure 3A; Supplementary Table S2). Subsequently, we constructed a protein-protein interaction (PPI) network for these 474 DEGs using STRING and Cytoscape (Supplementary Figure S1) and conducted enrichment analysis using ClueGO. We extracted two key modules (Figures 3B, C) and visualized all enrichment results (Figures 3D–F). Enrichment analyses indicated that these 474 DEGs were significantly enriched in 419 GO and 33 KEGG pathways, respectively (Supplementary Table S3), with the majority of pathways being associated with immune responses. The top 10 and 20 highly enriched GO and KEGG pathways are presented in Figures 3G, H, respectively.

### 3.4 Construction of telomere prognostic model in OS

**FIGURE 4:**
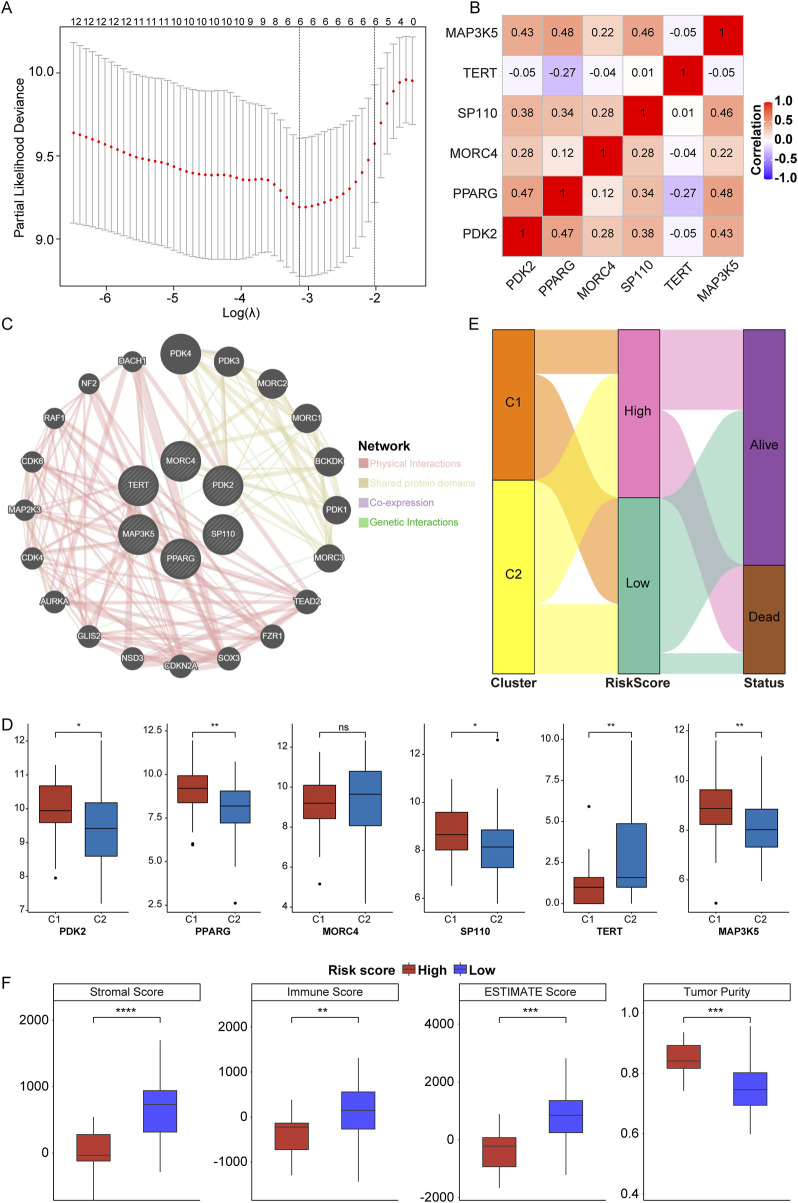
Construction of telomere prognostic model in OS. **(A)** The lambdas quality control plots of LASSO Cox regression analysis. **(B)**. The correlation among these six genes. **(C)** The co-expression relationship of six genes explored by Genemania. **(D)** The expression of *PDK2*, *PPARG*, *MORC4*, *SP110*, *TERT*, and *MAP3K5* in C1 and C2 subtypes. **(E)** Sanggi diagram showing the relationship between subtype, survival status, and risk score. **(F)** The stromal, immune, ESTIMATES scores and tumor purity in high- and low-risk group. *p < 0.05, **p < 0.01, ***p < 0.001, ****p < 0.0001, ns means no significance.

In accordance with the twelve prognosis-related PRGs (p < 0.0001), LASSO Cox regression analysis was performed to further identify prognostic PRGs, resulting in the selection of six genes: *PDK2*, *PPARG*, *MORC4*, *SP110*, *TERT*, and *MAP3K5* (Figure 4A, where the lambda value was minimized). Subsequently, the expression levels of these six genes were weighted using the LASSO Cox regression coefficients to develop a telomere prognostic risk score model: Risk Score = (*PDK2* × −0.04986244) + (*PPARG* × −0.04608845) + (*MORC4* × −0.22995413) + (*SP110* × −0.43002151) + (*TERT* × 0.19101697) + (*MAP3K5* × −0.16274433). As illustrated in Figure 4B, no significant correlation was observed among the six genes. The GeneMANIA network analysis indicated potential protein interactions between these six proteins and an additional twenty proteins (Figure 4C). Moreover, we discovered that *PDK2*, *PPARG*, *SP110*, and *MAP3K5* expressions were significantly increased, and *TERT* expression was significantly decreased in C1 subtype compared to C2 subtype (Figure 4D).

In the TARGET-OS cohort, patients were categorized into high- and low-risk groups based on the optimal intercept value (0.53818). The relationship between risk score and survival status across telomere subtypes was examined, confirming our hypotheses. Patients classified under the C1 subtype exhibited significantly better outcomes compared to those in the C2 subtype, with the majority of patients presenting a low-risk score (Figure 4E). This finding substantiates the effectiveness of the prognostic model. Furthermore, stromal, immune, and ESTIMATE scores were significantly elevated in the low-risk group, despite lower tumor purity (Figure 4F, low vs high). These results suggest that OS patients in the low-risk group demonstrate enhanced immune and stromal activity, indicating that the risk score model is associated with the TME.

### 3.5 Verification of telomere prognostic model in OS

**FIGURE 5:**
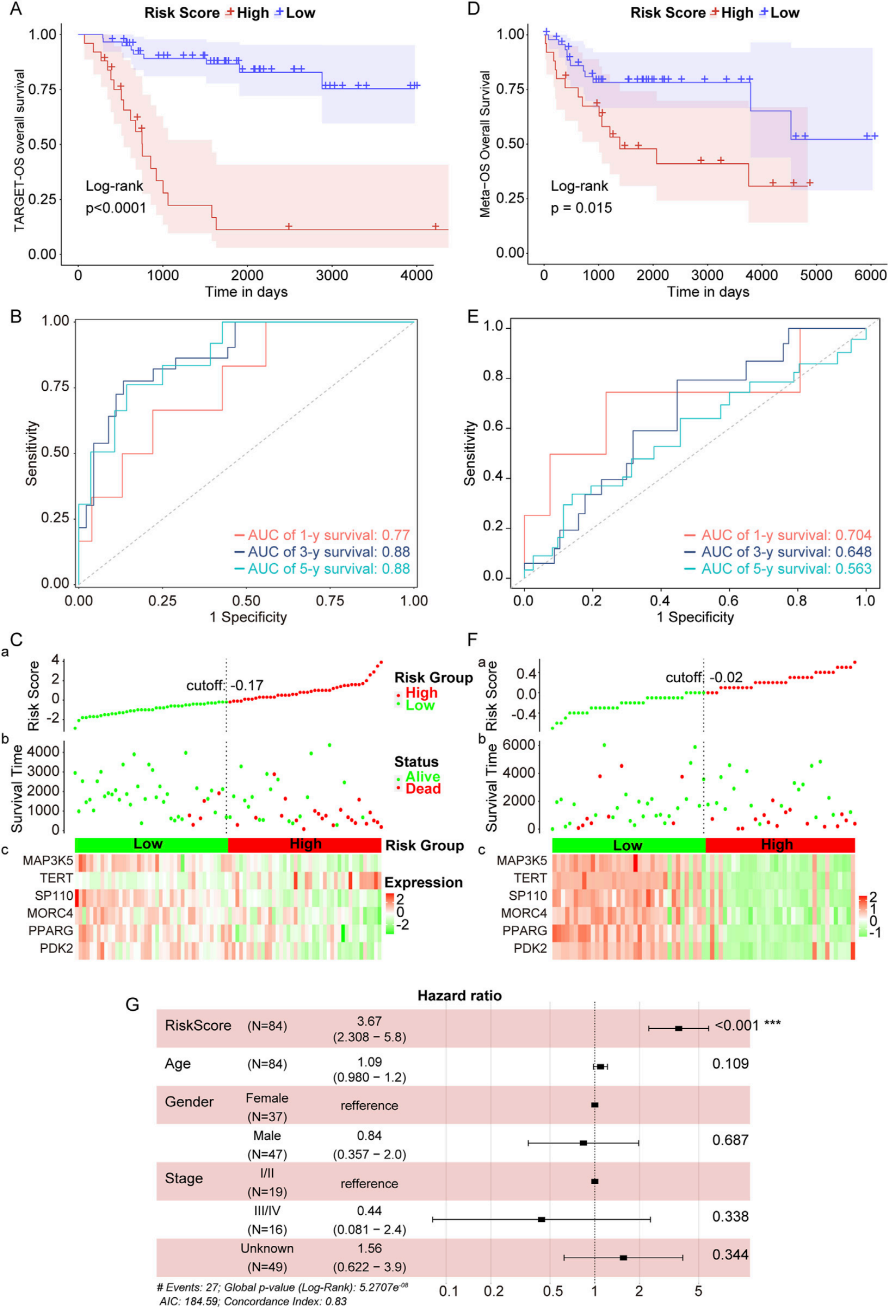
Verification of telomere prognostic model in OS. **(A)** The survival rate of patients in high- or low-risk group in the TARGET-OS cohort. **(B)** Time-dependent ROC analysis of TCGA- OS cohort. **(C)** Candidate gene expression and survival heatmap in TCGA-OS cohort. **(D)** The survival rate of patients in high- or low-risk group in the GEO-meta validation set. **(E)** Time-dependent ROC analysis of GEO-meta validation set. **(F)** Candidate gene expression and survival heatmap in GEO-meta validation set. **(G)** The association between the risk score and clinical characteristics was determined using multivariate Cox regression analysis. ***p < 0.001.

In the TARGET-OS cohort, we performed Kaplan-Meier (KM) analyses and found that the low-risk group exhibited superior prognostic performance compared to the high-risk group (Figure 5A). The area under the receiver operating characteristic (ROC) curves (AUC) was 0.77, 0.88, and 0.88 for 1, 3, and 5 years, respectively (Figure 5B). The expressions of six genes were negatively correlated with the survival of OS patients in the TARGET-OS cohort (Figure 5C). Additionally, in the GEO-meta validation set, which combined GSE16091 and GSE39055, OS patients were divided into high- and low-risk groups, revealing that patients in the high-risk group also exhibited inferior prognosis (Figure 5D). In this validation set, the AUC of the risk score was 0.704, 0.648, and 0.563 for 1, 3, and 5 years, respectively (Figure 5E), and the expressions of the six genes were negatively associated with patient survival (Figure 5F). As shown in Figure 5G, the risk score model emerged as an independent prognostic factor. Overall, these findings suggest that the telomere prognostic model serves as an independent prognostic factor for OS and demonstrates good predictive potential for OS patients.

### 3.6 Expression of six genes in different OS cell line

**FIGURE 6:**
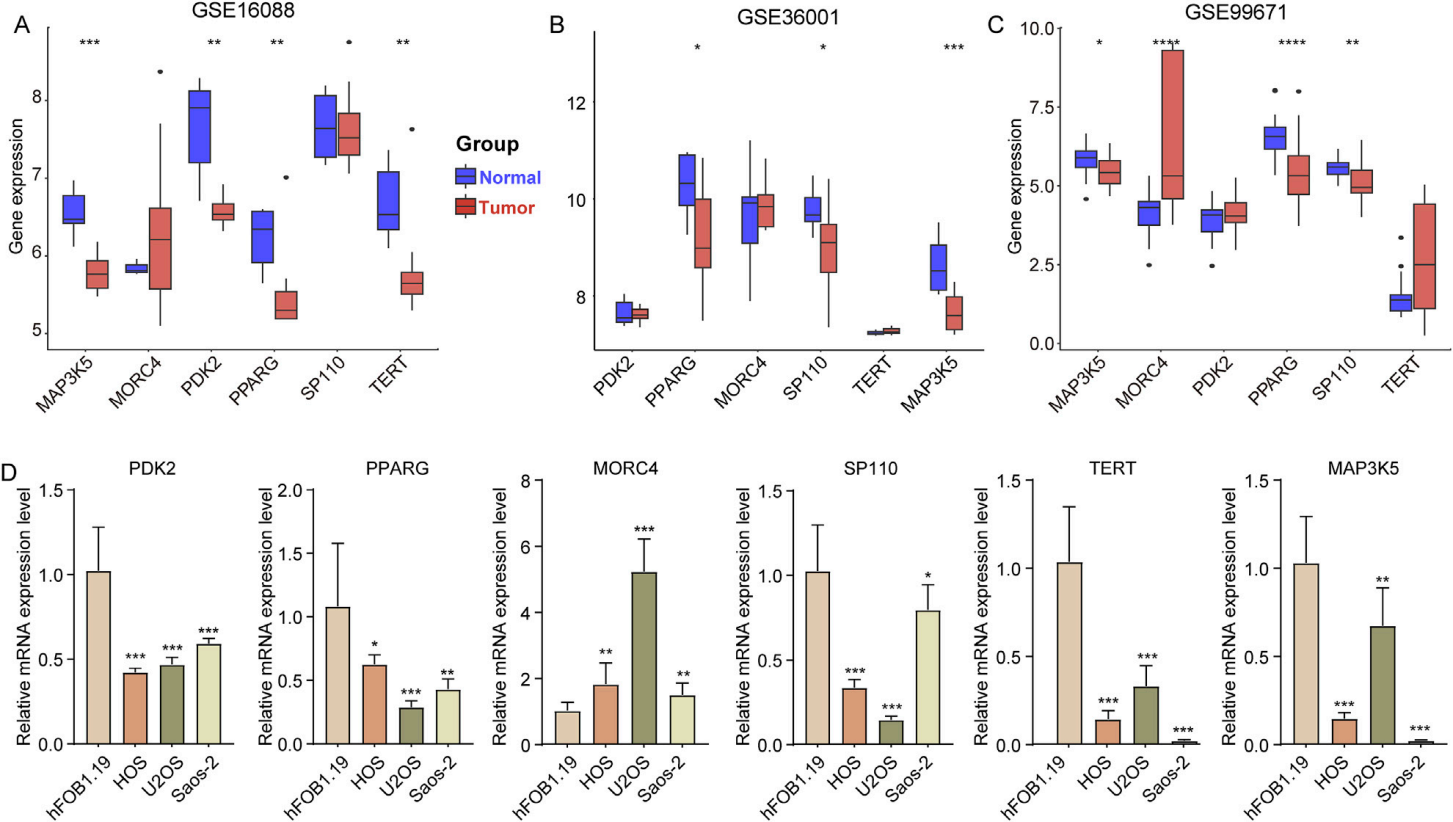
Expression of six genes in different OS cell line. **(A–C)** The expression of *PDK2*, *PPARG*, *MORC4*, *SP110*, *TERT* and *MAP3K5* in tumor and normal samples in the GSE16088, GSE36001, and GSE99671 datasets. **(D)** The expression of *PDK2*, *PPARG*, *MORC4*, *SP110*, *TERT* and *MAP3K5* in osteoblast cell line (hFOB1.19) and three OS cell lines (HOS, U2OS, and Saos-2). *p < 0.05, **p < 0.01, ***p < 0.001, ****p < 0.0001.

Finally, we analyzed the expression levels of *PDK2*, *PPARG*, *MORC4*, *SP110*, *TERT*, and *MAP3K5* in OS samples compared to normal bone samples. In the GSE16088 dataset, the expression of *MAP3K5*, *PDK2*, *PPARG*, and *TERT* was found to be downregulated in OS samples relative to normal bone samples (Figure 6A). Similarly, in the GSE36001 dataset, the expression of *PPARG*, *SP110*, and *MAP3K5* was reduced in OS samples compared to normal samples (Figure 6B). In the GSE99671 dataset, while the expression of *MAP3K5*, *PPARG*, and *SP110* was decreased, *MORC4* expression was increased in OS samples (Figure 6C, OS vs normal samples). Furthermore, when comparing the expression levels in three OS cell lines (HOS, U2OS, and Saos-2) to those in the osteoblast cell line hFOB1.19, we observed a significant decrease in the expression of *PDK2*, *PPARG*, *MORC4*, *SP110*, *TERT*, and *MAP3K5* (Figure 6D).

## 4 Discussion

OS is the most common primary bone tumor in humans, predominantly affecting children and adolescents (Marko et al., 2016). OS exhibits considerable heterogeneity due to its histological diversity and genetic instability, which contribute to its poor prognosis (Mutsaers and Walkley, 2014). Although several studies have identified various molecular subtypes of OS, significant heterogeneity and limitations remain (Guerin et al., 2016; Shu et al., 2022). Therefore, investigating the precise molecular subtypes of OS is essential for improving the survival rates of affected patients. Telomeres play a crucial role in the progression of OS, as cancer cells achieve unlimited replication by activating telomere-maintenance mechanisms (TMM), such as telomerase or the ALT pathway (Gao and Pickett, 2022). It has been reported that telomere shortening may act as a tumor suppressor by hindering cell growth and inducing widespread genomic instability, thereby facilitating cancer progression (Okamoto and Seimiya, 2019; Li et al., 2022). Additionally, telomere shortening could enhance the sensitivity of OS cells to apoptosis and ionizing radiation (Qiang et al., 2014; Li et al., 2015). Longer telomeres in leukocytes have been identified as a significant risk factor for the development of myeloproliferative neoplasms (Bao et al., 2020). Furthermore, telomeric events might influence the innate immune system and the tissue microenvironment. While telomere shortening appears to enhance systemic inflammation, it can also restrict immune function by limiting the proliferation of immune cells (Georgin-Lavialle et al., 2010). Thus, investigating telomere characteristics may offer novel targets for cancer treatment.

Based on the expression of 400 TRGs, OS was classified into two distinct molecular subtypes: C1 and C2. The survival rate of OS patients in subtype C2 was found to be inferior to that of patients in subtype C1. In comparison to C2, the C1 subtype exhibited increased stromal, immune, and ESTIMATES scores, along with decreased tumor purity. The DEGs between the C1 and C2 subtypes were highly enriched in immune-related pathways. Furthermore, we observed that, unlike C1, which demonstrates a clear prognostic advantage, various immune-related signaling pathways were significantly downregulated in C2. Additionally, the C1 subtype was significantly associated with a greater abundance of immune cells compared to the C2 subtype. These findings suggest that immune function in the C2 subtype may be inhibited relative to that in the C1 subtype.

In examining the effects of telomeres on the progression of OS, we identified six hub TRGs—*PDK2*, *PPARG*, *MORC4*, *SP110*, TERT, and *MAP3K5*—that were correlated with OS prognosis. Subsequently, we developed a telomere prognostic risk score model based on these six genes to predict OS outcomes. Previous studies indicate that the TRG risk model has the potential to predict the prognosis of patients with various types of cancer. For instance, Li et al. reported that a risk model constructed by ten TRGs could forecast outcomes for kidney cancer patients and may assist in the selection of treatment agents for this population (Li et al., 2022). A novel risk model constructed using eight TRGs was correlated with survival and prognosis of patients with oral squamous cell carcinoma (Yue and Yao, 2023). Our risk score model was related to the TME in OS, revealing that patients with a high-risk score had a poorer prognosis than those with a low-risk score. Thus, the telomere-related signature demonstrated significant potential as an independent prognostic factor for OS, showcasing a remarkable ability to forecast patient outcomes.

Pyruvate dehydrogenase 2 (*PDK2*) was highly expressed across various tumors and is closely associated with the malignant phenotypes of cancers (Sutendra et al., 2013; Sun et al., 2017). In the context of OS, inhibiting *PDK2* was found to slow tumor growth and progression (Cao et al., 2019). Given that PDK is a crucial regulator of glycolysis and oxidative phosphorylation (Li et al., 2023), we hypothesized that *PDK2* may influence the energy metabolism of osteosarcoma cells by modulating the balance between glycolysis and oxidative phosphorylation, thereby playing a role in the progression of OS. Peroxisome proliferator-activated receptor gamma (PPARG) belongs to a group of nuclear receptors responsible for regulating reproduction, metabolism, development, and immune responses (Montaigne et al., 2021; Stark et al., 2021). This receptor exhibits diverse biological functions and is crucial in the management of metabolism, inflammation control, atherosclerosis improvement, tumor inhibition, and immune modulation (Jabbari et al., 2021). Research showed that *PPARG* antagonists could abnormally activate the PI3K/AKT/mTOR and ERK/mTOR pathways in osteosarcoma cells and effectively inhibited the growth of OS *in vivo* and *in vitro* (Yuan et al., 2023). MORC family CW-type zinc finger 4 (MORC4) is a member of the highly conserved MORC family, it has been observed to be highly expressed in multiple cancer, such as colorectal cancer (Liang et al., 2023), breast cancer (Yang et al., 2019), and lymphomas (Liggins et al., 2007). Overexpression of *MORC4* significantly increases the expression of BCL-2 in breast cancer cells and increases their resistance to adriamycin, 5-fluorouracil and cisplatin (Luo et al., 2020). A recent study showed that baicalin is a potential anti-tumor drug that inhibits the tumorigenic behavior of breast cancer cells by reducing the expression of *MORC4* (Duan et al., 2019). Thus, we hypothesized that *MORC4*may influence OS progression and drug resistance. SP110 is part of the nuclear body protein family and is involved in immune response regulation. Although the specific role of SP110 in OS is not detailed in the context, it is known that immune response pathways can significantly impact tumor progression (Fan et al., 2024). The interaction of tumor cells with the immune system can influence tumor growth and metastasis. For instance, the expression of histocompatibility proteins and other immune-related factors has been associated with osteosarcoma development (Zhao et al., 2015), suggesting that SP110 may play a role in modulating the immune environment surrounding the tumor, potentially affecting its progression through immune evasion or modulation of inflammatory responses. Telomerase reverse transcriptase (TERT) is crucial for maintaining telomere length, which is often dysregulated in cancer (Dratwa et al., 2020). The dysregulation of *TERT* can contribute to genomic instability and promote tumorigenesis by allowing cancer cells to bypass normal cellular senescence mechanisms (Liu et al., 2024; Murugan et al., 2024). A pan-cancer analysis demonstrates that the expression variability of TERT is correlated with multiple cancer types and is associated with DNA methylation and immune cell infiltration (Xie L. et al., 2023). Inhibiting *TERT* has been shown to reduce the proliferation, motility, and metastasis of OS cells (Xie L. et al., 2023). Thus. *TERT* might affect the progression of OS by regulating telomere length. *MAP3K5* played a crucial role in regulating reactive oxygen species (ROS) (Ichijo et al., 1997). Activation of *MAP3K5* leaded to the production of pro-inflammatory cytokines, which was vital for the innate immune response (Matsuzawa et al., 2005). Notably, *MAP3K5* was downregulated in OS cells, where it inhibited cell growth and motility, promotes cell death, and generates ROS (Xie B. et al., 2023). Notably, the expression of *MAP3K5* was higher in OS compared to normal bone samples. Accordingly, MAP3K5 may affect the tumor microenvironment of OS by regulating ROS and inflammation-related signaling pathways, thereby affecting the occurrence and prognosis of OS.

In conclusion, we identified two different molecular subtypes based on TRGs, and constructed a telomere-related signature consisting of *PDK2*, *PPARG*, *MORC4*, *SP110*, *TERT* and *MAP3K5* in OS. Telomere prognostic model might be a critical biomarker for predicting prognosis, defining molecular subtypes, and describing immune cell infiltration in OS patients. However, the correlation between telomere and immunity needs to be further research at the cellular and molecular levels.

## Data Availability

The original contributions presented in the study are included in the article/Supplementary Material, further inquiries can be directed to the corresponding author.
