# Supplementary figures and images for: Identification of telomere-related gene subtypes and prognostic signatures in osteosarcoma
Source: Front Pharmacol. 2025 Feb 25;16:1545913. doi: 10.3389/fphar.2025.1545913 (PMC11893505; doi:10.3389/fphar.2025.1545913)

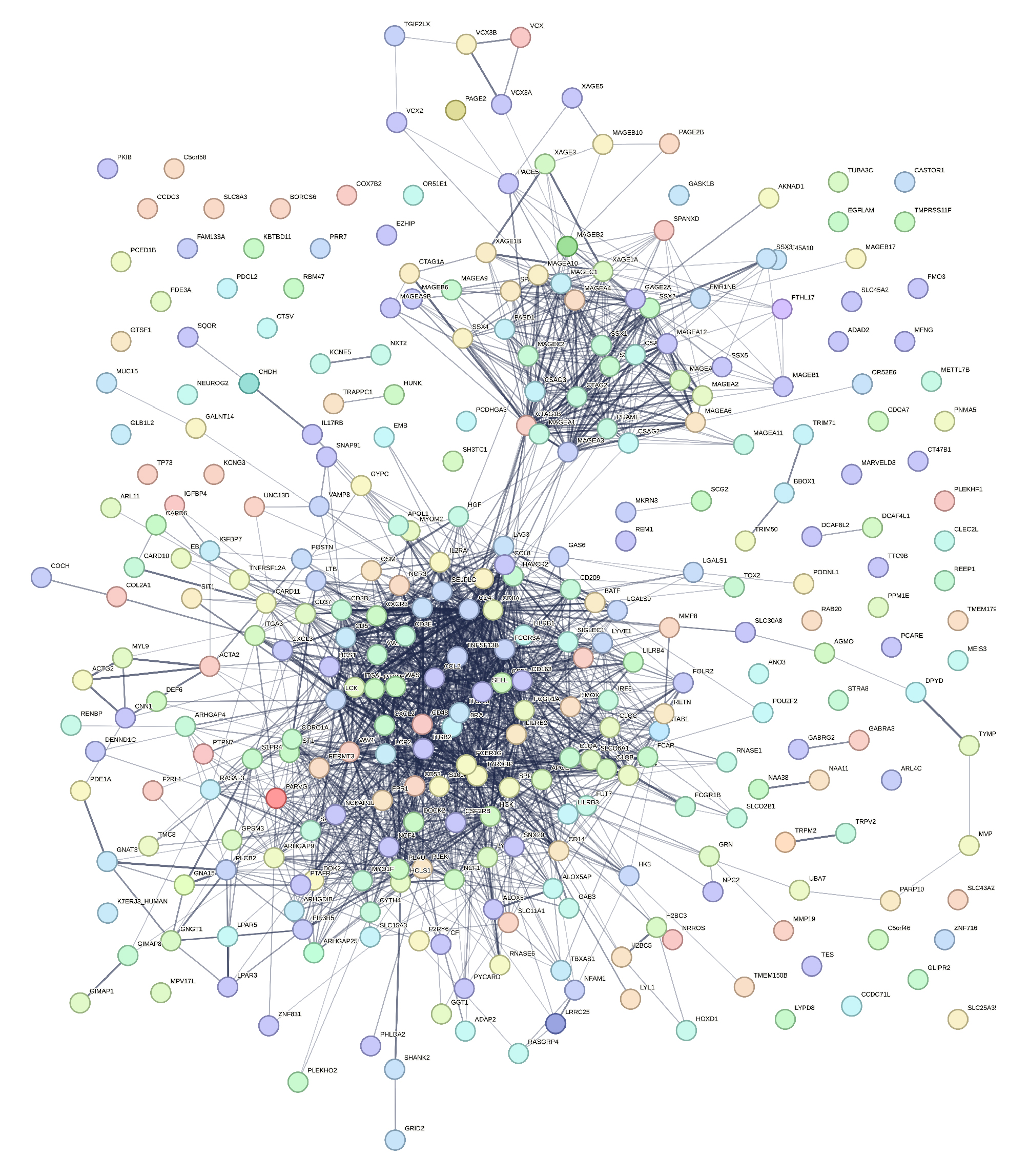

Supplement: Supplementary file 1 [file Image1.jpeg]
